# HDAC inhibitor protects chronic cerebral hypoperfusion and oxygen‐glucose deprivation injuries via H3K14 and H4K5 acetylation‐mediated BDNF expression

**DOI:** 10.1111/jcmm.15358

**Published:** 2020-05-06

**Authors:** Yao‐Ching Fang, Lung Chan, Jing‐Ping Liou, Yong‐Kwang Tu, Mei‐Jung Lai, Chin‐I Chen, Amelia Nur Vidyanti, Hsueh‐Yun Lee, Chaur‐Jong Hu

**Affiliations:** ^1^ Taipei Neuroscience Institute Taipei Medical University Taipei Taiwan; ^2^ Department of Neurology Shuang Ho Hospital Taipei Medical University New Taipei City Taiwan; ^3^ School of Pharmacy College of Pharmacy Taipei Medical University Taipei Taiwan; ^4^ TMU Biomedical Commercialization Center Taipei Medical University Taipei Taiwan; ^5^ Department of Neurology Wan Fang Hospital Taipei Medical University Taipei Taiwan; ^6^ Department of Neurology Faculty of Medicine Public Health and Nursing Universitas Gadjah Mada Yogyakarta Indonesia; ^7^ International Master/PhD Program in Medicine College of Medicine Taipei Medical University Taipei Taiwan

**Keywords:** HDAC, histone acetylation, histone deacetylase inhibitor, OGD, vascular dementia

## Abstract

Vascular dementia (VaD) is the second most common cause of dementia, but the treatment is still lacking. Although many studies have reported that histone deacetylase inhibitors (HDACis) confer protective effects against ischemic and hypoxic injuries, their role in VaD is still uncertain. Previous studies shown, one HDACi protected against cognitive decline in animals with chronic cerebral hypoperfusion (CCH). However, the underlying mechanisms remain elusive. In this study, we tested several 10,11‐dihydro‐5H‐dibenzo[b,f]azepine hydroxamates, which act as HDACis in the CCH model (in vivo), and SH‐SY5Y (neuroblastoma cells) with oxygen‐glucose deprivation (OGD, in vitro). We identified a compound 13, which exhibited the best cell viability under OGD. The compound 13 could increase, in part, the protein levels of brain‐derived neurotrophic factor (BDNF). It increased acetylation status on lysine 14 residue of histone 3 (H3K14) and lysine 5 of histone 4 (H4K5). We further clarified which promoters (I, II, III, IV or IX) could be affected by histone acetylation altered by compound 13. The results of chromatin immunoprecipitation and Q‐PCR analysis indicate that an increase in H3K14 acetylation leads to an increase in the expression of BDNF promoter II, while an increase in H4K5 acetylation results in an increase in the activity of BDNF promoter II and III. Afterwards, these cause an increase in the expression of BDNF exon II, III and coding exon IX. In summary, the HDACi compound 13 may increase BDNF specific isoforms expression to rescue the ischemic and hypoxic injuries through changes of acetylation on histones.

## 1 INTRODUCTION

Vascular dementia (VaD) is the most common of cognitive disorders in elderly persons after Alzheimer disease (AD). The causes of VaD are diverse and its pathophysiology is complicated.^1^, ^2^, ^3^, ^4^ Significant progress has been achieved in reducing stroke risk, but there remains a lack of effective treatment for VaD after stroke. Although numerous pre‐clinical drugs have been tested, currently, only the use of intravenous thrombolytic agents in the ultra‐acute stage^5^ is approved by the FDA for stroke treatment because the mechanisms underlying chronic ischemia and hypoxia are not fully understood. Chronic cerebral hypoperfusion (CCH), which is a major cause of VaD, has been shown to involve amyloid accumulation and inflammation.^1^ Further clarification of the role in these pathophysiologic changes play in VaD and the development of a related therapy are warranted.

Epigenetic modification plays an important role in the pathophysiology of many diseases. Histone deacetylases (HDACs), along with histone acetyltransferases (HATs), regulate chromatin remodelling and subsequent gene transcription by controlling the status of histone acetylation. Histone deacetylation removes acetyl groups from histones and induces condensed chromatin conformation contributing to the suppression of gene transcription.^6^ This phenomenon is involved in diverse physiological processes. In some brain disorders, such as ischemic stroke and AD, HDACs are dysregulated and play a role in the pathophysiology of these diseases.^7^, ^8^, ^9^, ^10^, ^11^ This suggests that HDACs might be potential targets for the treatment of brain disorders. Recently, HDAC inhibitors (HDACis) have been used in therapy for acute brain injury and neurodegenerative diseases.^12^

The administration of HDACis in the animal model of acute stroke was shown to reduce infarction size, suppress neuroinflammation and improve neurological deficits. It also decreased oedema and improved blood‐brain barrier (BBB) disruption by inhibiting NF‐κB activation and matrix metallopeptidase expression.^13^ Moreover, HDACis could be given either before or even 3‐h after ischemic injury.^14^ In addition to stroke, a prior study pointed out that pan‐HDACis (Trichostatin) could effectively improve outcome in the animal model of multiple sclerosis by mitigating the inflammatory response, demyelination, and nerve or axonal damage. However, the effects of HDACis during post‐stoke vascular cognitive impairment still needs to be investigated.

The manipulation of HDACis might serve as a novel therapeutic strategy to promote stroke recovery. For example, in a prospective human study, sodium valproate, which is a non‐specific inhibitor of HDAC9 activation, was associated with a reduced risk of recurrent ischemic stroke.^15^

Furthermore, knockdown of HDAC2 promoted the recovery of motor dysfunction by enhancing neuronal survival, increased neuroplasticity of surviving neurons, and reduced neuroinflammation. Meanwhile, overexpression of HDAC2 may worsen stroke‐induced functional impairment.^16^ All of these findings support the idea that inhibition or enhancement of HDACs exerts a protective or detrimental effect in ischemic diseases, respectively.

Histone acetylation decreases along with ageing. This can cause abnormal synaptic plasticity and subsequent hippocampus‐dependent memory decline.^17^, ^18^ The dysregulation of HDACs can also disrupt chromatin remodelling, which is associated with AD.^19^, ^20^ HDAC can regulate DNA expression and modify some proteins, such as amyloid precursor protein and amyloid‐beta; in other words, it has an essential role in AD pathogenesis.^21^ These data show that HDAC inhibition is involved in a variety of neuroprotective mechanisms in the ischemic brain and that HDAC inhibition is used as a therapeutic agent for the treatment of post‐ischemic brain damage.

HDAC inhibition increases neurotrophic factors, brain‐derived neurotrophic factor (BDNF) expression, and dendritic spine density during ethanol withdrawal.^22^ This implies that BDNF activation may be beneficial in synaptic plasticity or memory consolidation. BDNF, a secreted protein of the neurotrophin family, binds to two differential receptors, TrkB and p75, which is followed by activation of respective downstream signalling pathways.^23^, ^24^ In addition to playing a role in synaptic plasticity, BDNF is critical for long‐term potentiation, which makes it critical in learning and memory. One major study indicated that exogenous BDNF expression protects brain slices against oxygen‐glucose deprivation (OGD).^25^ Furthermore, lipophilic statin pitavastatin treatment exerts its neuroprotection in cultured cerebral neurons after OGD involving the BDNF‐TrkB signalling pathway.^26^ The regulation of BDNF expression has been proved to be associated with numerous neurodegenerative diseases, mood disorders and ageing but is not fully understood in relation to VaD diseases.

CCH is closely associated with vascular cognitive impairment and VaD.^27^, ^28^, ^29^ We have established a mouse model to induce CCH by occlusion of the bilateral common carotid artery (modified BCCAO). This model shows cognitive decline, hippocampal atrophy and chronic neuroinflammation. By contrast, OGD is widely used to study the cell model of stroke and is a simple but useful technique for not only elucidating the molecular mechanisms of ischemic injury but for developing neuroprotective drugs. In this study, we synthesized a series of 10,11‐dihydro‐5H‐dibenzo[b,f]azepine hydroxamates as HDACis. These compounds were designed to confer the structural characteristics of the HDAC, including the zinc‐binding domain, linker domain and hydrophobic‐capping group. We screened and synthesized these HDACis by using OGD and identified compound 13, which was tested in CCH animals. This study also explored the possible mechanisms of VaD, focusing on the role of BNDF.

## 2 MATERIALS AND METHODS

### 2.1 Animals

Male C57bl/6j mice (16 weeks old, weighing 25‐35 g, Bio‐Lasco Taiwan Co., Ltd) were used for all the experiments and placed under controlled temperature (22 ± 1°C) and humidity (55% ± 10%) with a 12‐hour light/dark cycle (lights on at 07:00). Food and water were given ad libitum to all mice throughout the experiments. Animal care and experimental procedures in this study were performed in accordance with the guidelines for the Care and Use of Laboratory Animals from the Ethics Committee of Taipei Medical University.

### 2.2 BCCAO surgery

The modified‐BCCAO surgery was performed by ligating the common carotid artery (CCA), with some modification to a previous procedure.^30^ Briefly, a sagittal midline incision (approximately 1 cm in length) was made to expose the parietal skull to measure the baseline CBF using laser doppler flowmetry. Then, a cervical midline neck incision (approximately 1 cm in length) was made, both CCAs were carefully separated, and the right CCA was occluded. One week later, the procedure was repeated for the left CCA. The steps were similar, except there was transient ligation of the left CCA for 30 minutes by tightening the silk sutures. The CBF was measured to ensure that there was a reduction of CBF by 80%–90% from the baseline. The procedure of sham surgery was similar to BCCAO but involved no ligation of both CCAs. DMSO or compounds were given intraperitoneally (25 mg/mL) once every 2 days for either 1 or 3 months, beginning 2 days after CCA ligation. The mice were grouped as sham‐control (n = 12), DMSO with BCCAO (n = 12) and compound 13 or SAHA with BCCAO (n = 12).

### 2.3 Cell culture

The human neuroblastoma cell line SH‐SY5Y was cultured in Dulbecco's modified Eagle medium/F12 (Thermo Fisher Scientific) containing 10% foetal bovine serum at 37°C in a humidified atmosphere containing 95% air and 5% CO_2_. Cells were rendered quiescent by serum starvation for 24 hours before all experiments. Hypoxic injury was induced by hypoxia for 6 hours (ie incubation in OGD medium containing 2.3 mmol/L CaCl_2_, 5.6 mmol/L KCl, 154 mmol/L NaCl, 5 mmol/L Hepes, and 3.6 mmol/L NaHCO_3_ [pH 7.4] and under an atmosphere of 5% CO_2_, 95% N_2_ and <0.1% O_2_). The DMSO or HDAC inhibitor was treated with OGD medium. The neutralizing antibody of BDNF (1, 3 or 10 µg/mL) was treated for 24 hours before OGD condition.

### 2.4 3‐(4,5‐Dimethylthiazol‐2‐yl)‐2,5‐diphenyltetrazolium bromide (MTT)

Following incubation and treatment with different HDACis under control or H conditions for differential time, cell viability was tested by incubation with MTT reagent (Sigma‐Aldrich) at a final concentration of 0.5 mg/mL and a temperature of 37°C for 4 hours. The optical density of the purple MTT formazan product was measured at 570 nm using an enzyme‐linked immunosorbent assay reader. The absorbance of cells transfected with NC was regarded as indicating 100% viability.

### 2.5 Real‐time quantitative polymerase chain reaction (qPCR)

The expression of genes was examined through real‐time qPCR using G3PDH as internal control. The extracted total RNA was then reverse transcribed into cDNA. The reverse transcripts containing exon (I‐IX, II‐IX, III‐IX, IV‐IX or IX) were measured.

### 2.6 Western blot analysis

Proteins were resolved on the basis of molecular weight through electrophoresis on 8%, 10% and 12% polyacrylamide gels, followed by transfer to a polyvinylidene difluoride membrane. The membrane was blocked and incubated with antibodies for acetylated proteins (ac‐H3 or ac‐H4), neurotrophic factor (BDNF) or beta‐actin. Protein levels were analysed using an enhanced chemiluminescence detection kit (GE Healthcare).

### 2.7 Immunohistochemistry

At 3 months after the BCCAO or sham surgery, the mice were anaesthetized and intracardially perfused with phosphate‐buffered saline (PBS), followed by 4% paraformaldehyde. The brains were removed, post‐fixed overnight in 4% paraformaldehyde at 4°C, and stored in 30% sucrose in 0.1 mol/L PBS (pH 7.4). Serial coronal sections (30 μm) that spanned from the anterior of the corpus callosum (bregma 0.26 mm) to the anterior of the hippocampus (bregma 0.94 mm; adjusted according to the mouse brain atlas)^31^ were cut using a cryostat. Coronal sections were treated with 3% H_2_O_2_ in 0.01 mol/L PBS and pre‐incubated in 5% normal donkey serum. Subsequently, the sections were incubated in a primary Ab solution overnight at 4°C. After washing, the samples were incubated in a secondary Ab solution containing donkey anti‐rabbit or donkey anti‐goat biotinylated IgG (1:200, Zhongshan Biotechnology) for 1 hour at RT. Finally, the sections were incubated in HRP‐streptavidin (1:200, Zhongshan Biotechnology) for 1 hour at RT, and the colour was developed using the conventional reaction of diaminobenzidine with H_2_O_2_. All images were acquired using fluorescence microscopy (Olympus IX70) or light microscopy (Olympus).

### 2.8 BDNF promoter chromatin immunoprecipitation assay (ChIP)

A chromatin immunoprecipitation assay was used to study the interaction between intracellular DNA and protein. In this study, we used a ChIP assay kit (Abcam) to survey the relationship between the target gene promoter and the acetylated histone residue. Briefly, after cross‐linking the DNA and proteins by adding formaldehyde into the medium, the efficiency of formaldehyde was quenched by adding 0.125 mol/L glycine. Then, the DNA in cells was sheared into the average fragment (size: 200‐1000 bp) through lysis buffer and sonication. The antibody for the acetylated protein was used to immunoprecipitate the target DNA, and the DNA was eluted for real‐time PCR, ChIP or sequencing.

## 3 RESULTS

### 3.1 HDACi protects SH‐SY5Y cells against OGD injury

**Figure 1:**
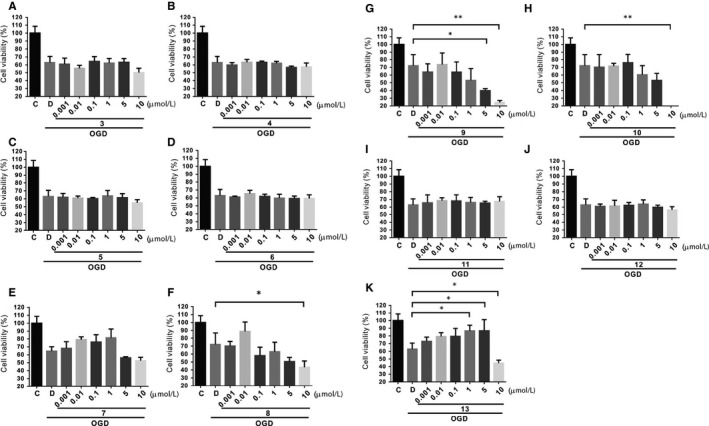
SH‐SY5Y neuroblastoma cells were subjected to OGD (hypoxia) injury in the presence of different concentrations of DMSO (control) and/or compounds 3‐13. A‐K, Relative cell viability was assessed through MTT assays. Data are presented as the mean ± standard error of the mean (SEM) of four experiments. *P < 0.05 compared with the group treated with DMSO under OGD condition

To examine the possible protection or injury attributable to treatment with compounds 3‐13 under the OGD condition, SH‐SY5Y cell viability was evaluated with an MTT assay. Cell viability was significantly reduced in the OGD condition in each group compared with the control group. In addition, the data showed that cell viability decreased significantly in cells treated with the tested compounds at 10 μmol/L (8:43.47% ± 3.93%, 9:24.51% ± 1.27%, 10:17.23% ± 1.17%, 13:44.74% ± 1.43%) vs DMSO (62.93% ± 3.89%; *P* < 0.05 for each group [n = 4]) (Figure 1F‐H,K). Furthermore, cells treated with compound 9 at 5 μmol/L (39.98% ± 1.21%) also decreased the cell viability compared with those in the DMSO control group (Figure 1G). However, while cell viability decreased at higher concentrations of some tested compounds, there were no changes in cells treated with compounds 3‐7, 11 or 12, compared with the DMSO group (Figure 1A‐E,J). In short, MTT activity of SH‐SY5Y cells following OGD‐induced injury was decreased by treatment with the synthesized compounds. The MTT activity dramatically increased in cells treated with compound 13 at 1 or 5 μmol/L (86.72% ± 3.60% or 86.85% ± 7.19%) in conditions of OGD (Figure 1K), and as a result, compound 13 was selected for further investigation.

### 3.2 Western blotting analysis identifies BDNF up‐regulation by HDACi (compound 13) in the neuronal cells under OGD condition and hippocampus of ischemic brain

**Figure 2:**
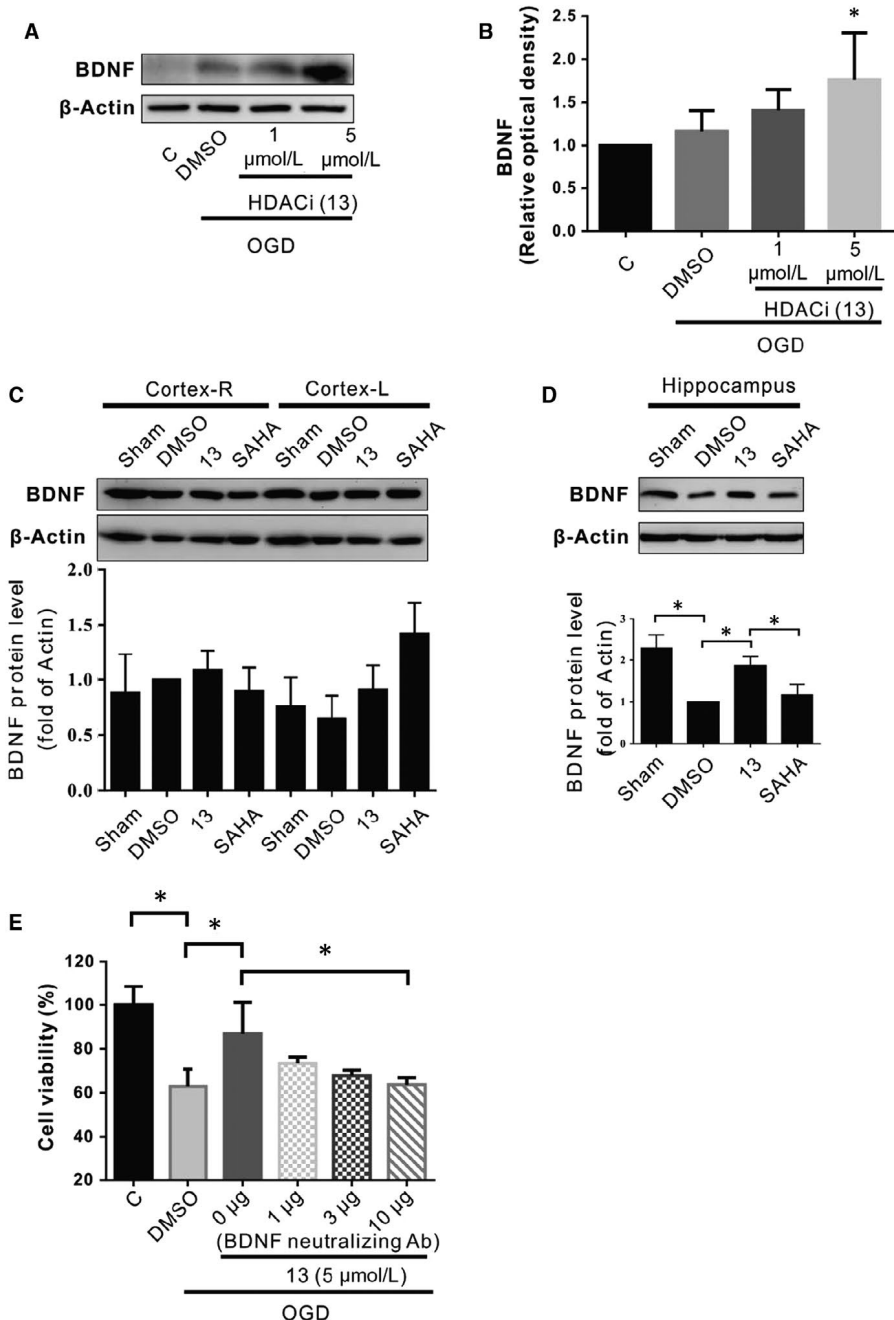
Effect of compound 13 on the protein levels of BDNF in the SH‐SY5Y cells under OGD condition (A‐B), cortex (right cortex, CR; left cortex, CL) (C) or hippocampus (H) (D) of CCH mice. The protein levels of BDNF were measured after BCCAO for 3 mo. β‐actin served as the loading control. The densities were normalized to β‐actin, and each bar represents the mean ± SEM of four independent experiments (n = 4, *P < 0.05, **P < 0.01 vs indicated group). The viability of cells treated with compound 13 in the presence or not of a neutralizing antibody of BDNF was noted. E, The cell viability was examined through MTT assay in cells exposed to OGD treated with compound 13 in the presence of a neutralizing antibody of BDNF (1, 3 or 10 µg/mL). Data are presented as the mean ± SEM of four experiments (n = 4, *P < 0.05 vs indicated group)

To test the underlying mechanism of the protective effect of HDACis in cognitive functioning, hippocampal volume (data not shown) and cell viability (Figure 1), we next clarified which molecules were involved in these processes. As can be seen from Figure 2, the content of BDNF in hypoxic cells was slightly increased, with compound 13 at 1 μmol/L, and the level of BDNF protein significantly increased at 5 μmol/L by treatment of compound 13 under the OGD condition for 24 hours compared with the DMSO group. Compound 13 (5 μmol/L) also effectively protected cells from hypoxia (Figure 2A,B). We found that the increase in BDNF levels in hypoxic cells with compound 13 was a potential mechanism providing neuroprotective effects against OGD. Therefore, we also considered whether compound 13 can increase the protein levels of BDNF in a CCH brain to protect against VaD. Although compound 13 does not significantly increase BDNF content on the left or right side of the cortex in a CCH mouse (Figure 2C), it is noteworthy that in the hippocampus, compound 13 significantly increases the protein levels of BDNF. As we know, suberoylanilide hydroxamic acid (SAHA) has a protective effect on ageing animals, and its effect is related to the increment of trophic factors.^32^ However, in our CCH animals, SAHA did not increase BDNF content under CCH‐induced injury (Figure 2C,D). The data showed that BDNF, a potent autocrine/paracrine neurotrophic factor, has a neuroprotective effect, which is induced by HDAC inhibitor, on ischemia/OGD injuries. We examined the possibility that treatment with HDACis might modulate BDNF expression and proved that BDNF expression was significantly up‐regulated in SH‐SY5Y cells subject to OGD with respect to mRNA expression and the protein level of such expression. Furthermore, to clarify that the effect of compound 13 was mediated through BDNF activation, we examined whether cell viability in the up‐regulation of BDNF by compound 13 protects cells from OGD. Specifically, SH‐SY5Y cells were treated with compound 13 in the presence of differential concentrations of the neutralizing antibody of BDNF (1, 3 or 10 µg/mL). We found that treatment of the BDNF antibody (10 µg/mL) significantly reversed the protective effect of compound 13 in cell viability under OGD conditions compared with cells not treated with the BDNF antibody (Figure 2E). Thus, BDNF might exert its role in mediating the protective role of HDAC inhibitor under hypoxia.

### 3.3 HDACi upregulates protein levels of H3K14 or H4K5 acetylation in OGD cells

**Figure 3:**
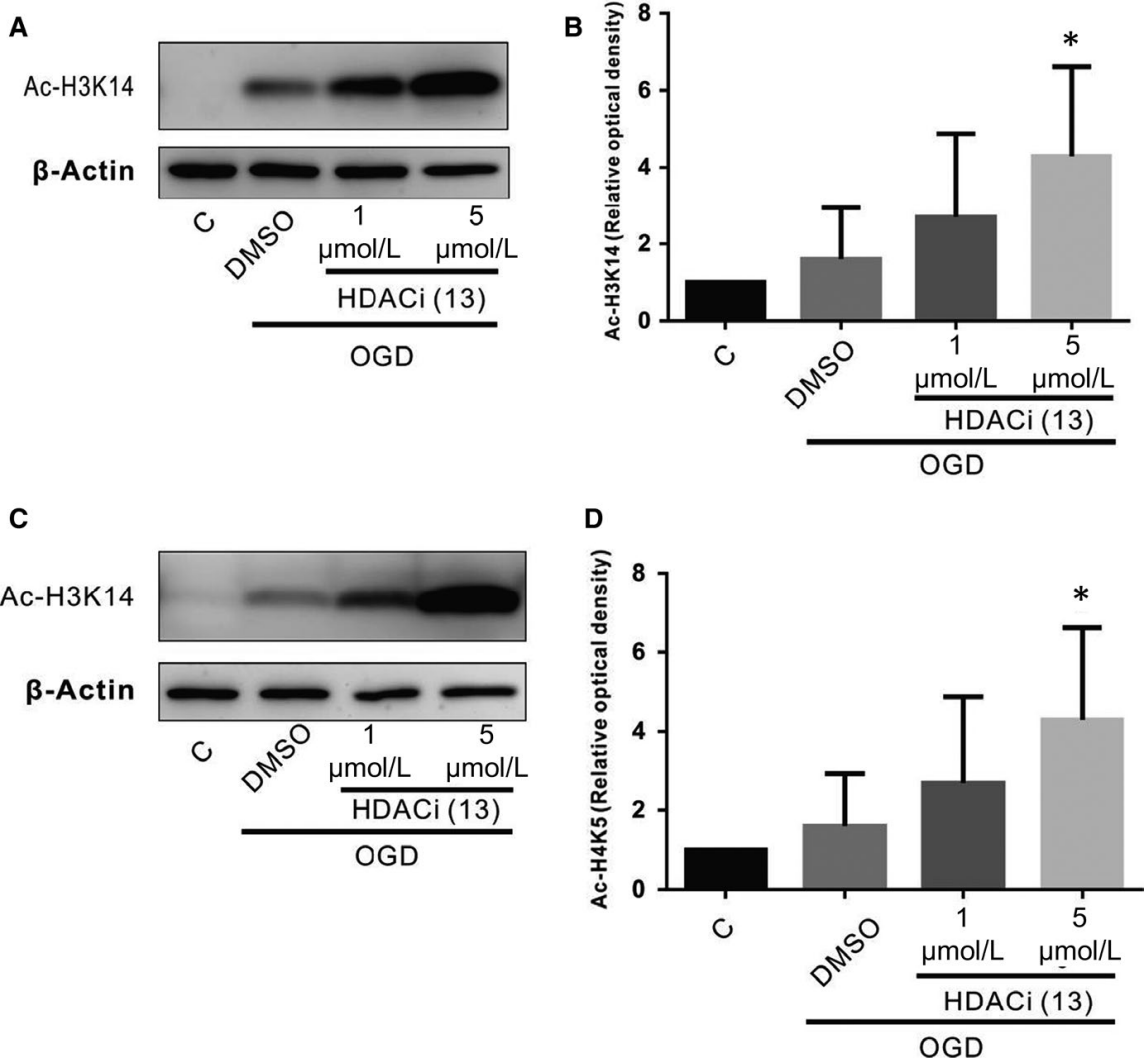
Effect of compound 13 on histone acetylation (H3K14 or H4K5) in the cells exposed to OGD injury. The levels of histone acetylation (Ac‐H3K14 (A, B) and Ac‐H4K5 (C, D)) were studied after OGD for 24 h with the Western blotting analysis. β‐actin served as the loading control. The densities were normalized to β‐actin, and each bar represents the mean ± SEM of four independent experiments (n = 4, *P < 0.05, **P < 0.01 vs indicated group)

We further showed that the increased expression of BDNF induced by compound 13 could be because of the activation of the endogenous TrkB receptor. Data have shown that HDACis can significantly increase the protein levels of BDNF in the presence of cellular hypoxia and the CCH mouse model. However, we studied this phenomenon more closely to determine which lysine residue acetylation to increase, and on which histone, to achieve this effect. According to our literature review, histone acetylation could occur on histone 3 (K4, K9, K14, K23, K27, K36, K79…) or histone 4 (K5, K8, K12, K16…). Our unpublished data showed that compound 13 suppresses class I HDAC (eg HDAC1, HDAC2 or HDAC8) activity, and another major study found that the acetylation of H3K14 and H4K5 are related to class I HDACis.^33^ Thus, we have been suggested that compound 13 might increase the acetylation of H3K14 and H4K5. The data here showed that the expression of acetylated H3K14 and acetylated H4K5 was not significantly changed in cells after OGD compared with those of cells in the control group. However, treatment with compound 13 (5 μmol/L) significantly increased the protein levels of acetylated H3K14 (Figure 3A,B) and acetylated H4K5 (Figure 3C,D) compared with the DMSO group (*P* < 0.05). There was no significant difference in acetylated H3K14 and acetylated H4K5 expression between the compound 13 (1 μmol/L)‐treated groups and the DMSO group.

### 3.4 HDACi upregulates *BDNF* promoter 2 around H3K14 and promoter 2 or 3 around H4K5

**Figure 4:**
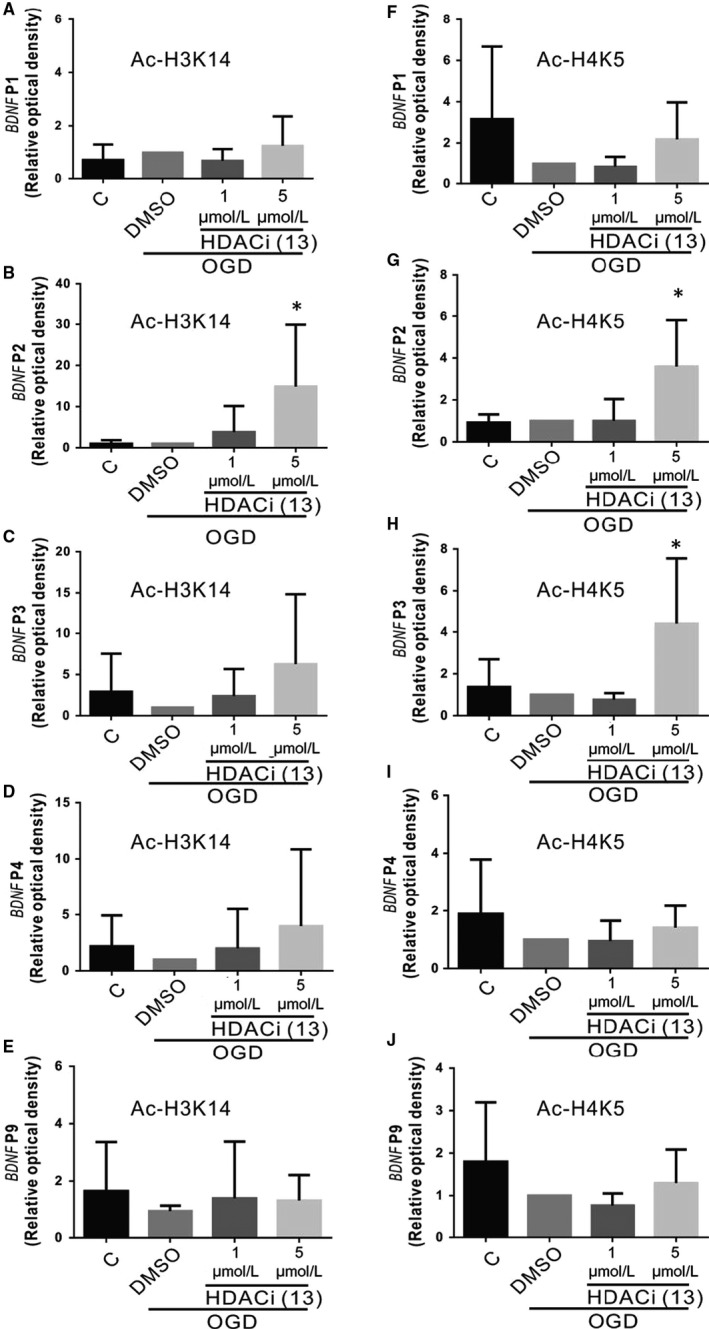
Effect of compound 13 on the *BDNF* promoter (I, II, III, IV or IX) around H3K14 or H4K5 in the cells exposed to OGD injury. A‐J, The expression of *BDNF* promoters was studied with chromatin IP, followed by qPCR analysis after OGD for 24 h. Fold expression was normalized to input control, and each bar represents the mean ± SEM of four independent experiments (n = 4, *P < 0.05, **P < 0.01 vs indicated group)

Histone acetylation increases transcription. Furthermore, neuroscientists have argued that the protective effect of HDACis in neurodegenerative diseases through incremental acetylation results in enhanced transcription of memory‐related genes. Data also show that HDACis can increase histone acetylation, followed by incremental increases of BDNF levels, which protect from OGD‐induced injury. However, the gap between histone acetylation and the protein levels of BDNF remains to be studied. Here, we used chromatin immunoprecipitation (IP) to investigate which *BDNF* promoter expression was increased around H3K14 or H4K5 after acetylation with HDACi treatment. The *BDNF* gene consists of eight 5′ non‐coding exons (exon I to VIII) and one 3′ coding exon (exon IX). One of the eight non‐coding exons conjugates with exon IX to form transcripts after RNA alternative splicing. Timmusk and colleagues^34^ indicated that not all *BDNF* transcripts would be transcripted according to neuronal activity. Thus, we proposed that histone acetylation might allow the transcription factor to bind to a differential *BDNF* promoter and proceeded to study *BDNF* promoter expression with qPCR under hypoxic conditions with HDACis. The data showed that the expression of *BDNF* P1 around H3K14 was slightly increased, but not significantly in cells treated with compound 13 compared with the DMSO group (Figure 4A). However, H3K14 acetylation by compound 13 increased the expression of *BDNF* P2 (Figure 4B). There were no changes in *BDNF* P1, P3, P4 or P9 expression in the cells treated with compound 13 (Figure 4A,C‐E). The expression of *BDNF* P2 and P3 also increased around H4K5 in compound‐13‐treated OGD cells (Figure 4G,H).

### 3.5 HDACi increases the expression of *BDNF* exon II, III and IX

**Figure 5:**
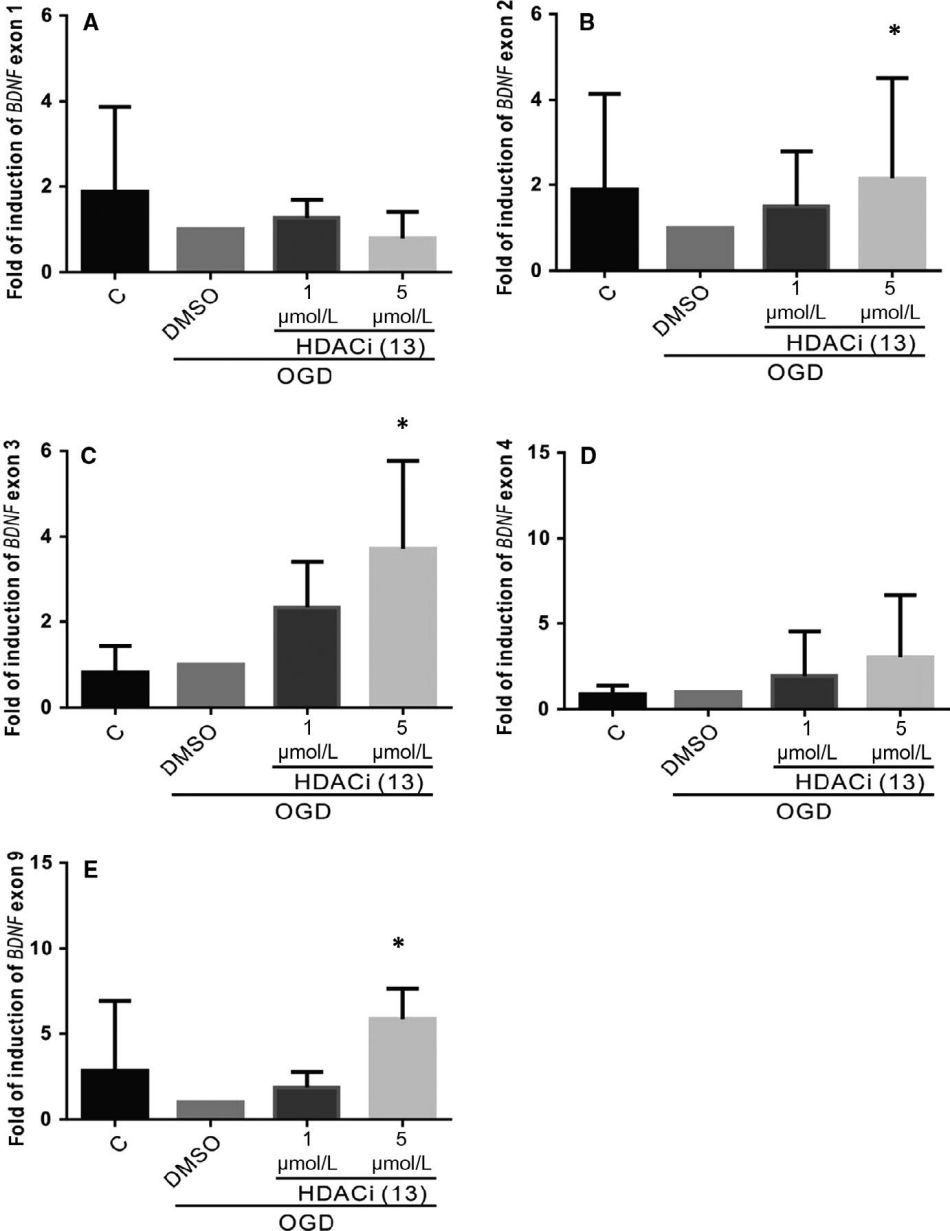
Effect of compound 13 on *BDNF* exon (I‐IX, II‐IX, III‐IX, IV‐IX or IX) in the cells exposed to OGD injury. A‐E, The expression of *BDNF* exons was studied with qPCR analysis after OGD for 24 h. The fold expression was normalized to GAPDH, and each bar represents the mean ± SEM of four independent experiments (n = 4, *P < 0.05, **P < 0.01 vs indicated group)

We further proved that the increased expression of *BDNF* P1 and P2 around H3K14 and of *BDNF* P2 and P3 around H4K5 resulted in the increase of relative transcripts. We designed the forward primer to complement one of the non‐coding exon sequences and the reverse primer to complement exon IX. Differential *BDNF* transcript expression was studied subsequently using qPCR. The data showed that the expression of *BDNF* exon II‐IX, III‐IX or IX could be increased (Figure 5B,C,E) but that of exon I‐IX or IV‐IX could not in the compound 13 group (Figure 5A,D).

## 4 DISCUSSION

**Figure 6:**
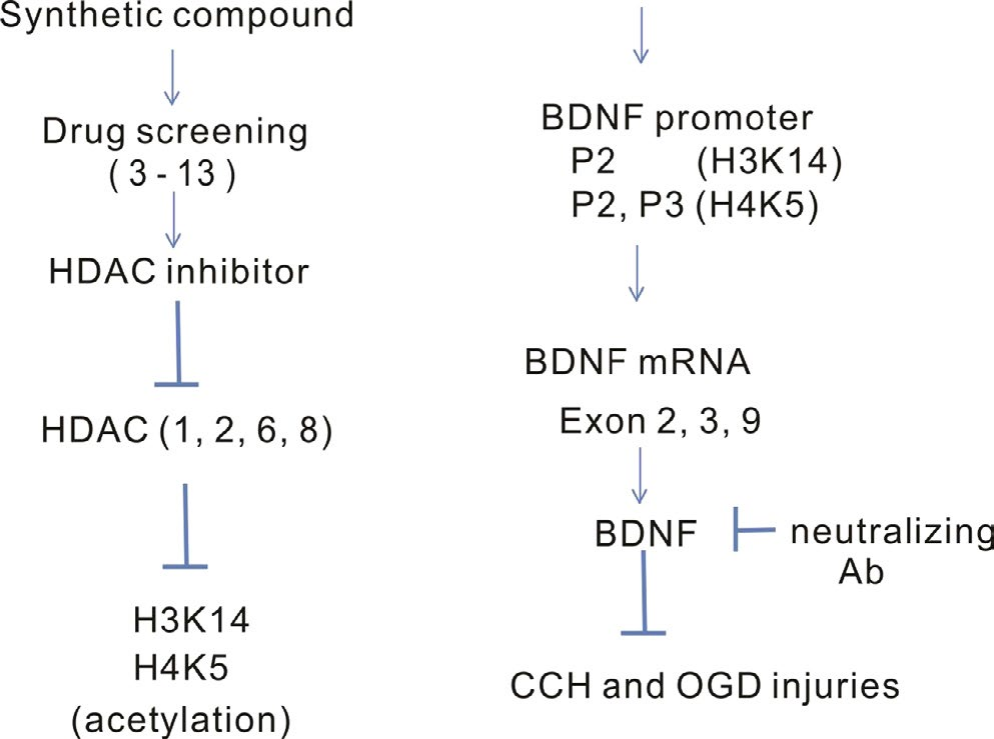
A proposed mechanism by which HDACi protects from injuries via BDNF expression

This is, to our knowledge, the first study to apply HDACis in the treatment of CCH animals and examine the molecular mechanisms in which BDNF is involved. One HDACi in particular, compound 13, increases the acetylation status in histones 3 and 4, which further react with specific promotors of *BDNF* and up‐regulate its protein levels (Figure 6).

HDACis usually comprise three structural features: a cap, linker and zinc‐binding group. We utilized the diphenyl core as the cap, and various linkers to connect the central core to the hydroxamic acid motif, which yielded a series of diphenyl hydroxamates. We synthesized different HDACis (compounds 3‐13) and modified their structures using SAHA. The reason we used SAHA as the base is because studies on SAHA have documented its promotion of anti‐apoptosis and anti‐inflammation in ischemic animals. However, no study has reported the effects of SAHA itself or its derivatives on the cognitive functioning or underlying mechanisms of the CCH model.

A considerable body of literature has indicated that HDACis have a protective effect on ischemia animals and hypoxic cells, but there is a lack of research on CCH‐induced VaD animals, the underlying mechanism of HDACis, and confirmation of the reaction between HDACis and the target genes. Evidence has indicated that HDAC inhibition works both transcriptionally and non‐transcriptionally to increase the expression of neurotrophic factors and stabilize microtubule proteins, respectively, which then increase vesicular transport.^12^ Compound 13 was selected because it was superior among HDACis in the OGD experiments (Figure 1). In this study, we focused on the effects of the transcriptional regulation of HDACis, but we cannot exclude the possibility that compound 13 achieved its effect by regulating non‐histone targets, which may include proteins involved in cytoplasmic signalling, hormone receptors, transcription factors or cytoskeletal proteins.^31^, ^35^, ^36^, ^37^

HDACis may be used to treat various brain diseases based on three possible mechanisms: (a) by reducing the anti‐inflammatory effects involving inflammatory factors, such as TNF‐alpha or IL‐1beta,^38^ (b) by reducing the neurotoxicity of β‐amyloid‐beta and other neurotoxic molecules,^39^ or (c) by increasing neuroprotective proteins such as glial cell‐derived neurotrophic factor or BDNF.^40^ Numerous studies have shown that the administration of HDACis increased the acetylation and transcription of some memory‐related genes and led to enhancement of hippocampus‐dependent memory.^41^, ^42^, ^43^ For example, HDACi (sodium butyrate) treatment also increased the phosphorylation of the cAMP‐response element‐binding protein (CREB) and BDNF after ischemic injury.^43^ However, the underlying mechanism of these HDACis remained unclear. Moreover, evidence suggests the screen of HDACis from the in‐house epigenetic library could modulate BDNF expression and neurogenesis‐related neurite outgrowth in human neural progenitor cells.^44^ Here, we demonstrated that the *BDNF* gene responded to epigenetic regulation, particularly HDACis and specifically hydroxamic acid‐based HDACis, under CCH and OGD conditions (Figure 2). Therefore, the compound obtained from our screening may provide a new treatment option for neurodegenerative diseases. Contrary to our data, some HDACis can inhibit BDNF expression, dendritic spine density and the excitatory quantal transmitter released in the neurons of the hippocampal CA1 region.^45^ In summary, the inhibition of HDAC resulted in changes of expression levels of BDNF, but the specific effects of HDACis on BDNF need further investigation.

The protective effects of BDNF in VaD are supported by the following evidence. L‐butylphthalide betulinic acid, found in the bark of plants, is used as an anti‐inflammatory drug,^46^ and dehydroepiandrosterone is an endogenous steroid hormone and functions in the biosynthesis of sex steroids.^47^ Both of these increased the expression levels of BDNF and cured the cognitive impairment measured by behavioural analysis in the BCCAO‐induced VaD rat model. In addition to drug delivery, both involuntary exercise induced by functional electrical stimulation and voluntary exercise increased the number of BDNF‐positive cells in the hippocampal CA1, CA2, CA3, and dentate gyrus regions and alleviated cognitive deficits in ischemic rats.^48^ In vitro data also showed that HDACi‐valproic acid and trichostatin A increased neuroprotection and BDNF expression.^49^ In short, the functions of BDNF in neurodevelopment, neurite outgrowth, synaptic plasticity and neuroprotection have been well established. In this study, we further confirmed the neuroprotective effects of BDNF through inhibition of BDNF using a BDNF‐neutralizing antibody (Figure 2E).

The knowledge gap on the relationship between HDACis and BDNF expression involving HDAC activity and acetylation of lysine residue on histones needs to be closed. Many studies have found that HDACis mitigate different HDAC isoform activities. Analyses of the HDAC isoform activity profiles of SAHA have revealed that SAHA inhibits mainly class I HDACis (HDAC1, 2, 3 and 8) and that subsequent neuroprotection in ischemic or aged animals was found.^15^, ^50^, ^51^, ^52^ HDAC6, belonging to the members of the class II HDAC family (HDAC IIa‐4, ‐5, ‐7, or ‐9, IIb‐6 and ‐10), is also inhibited by SAHA.^53^ These pre‐clinical data suggest that the inhibition of class I HDAC isoforms could be an avenue for the treatment of neurodegenerative disease^54^; similarly, our unpublished data showed that inhibition of HDAC1, 2 or 8 activity by compound 13 effectively mitigated CCH‐induced cognitive impairment. Furthermore, Johnsson et al^55^ demonstrated that HDACis working on HDAC class I can increase the acetylation of H3K14, which is consistent with our finding that compound 13 increases the acetylation of both H3K14 and H4K5 (Figure 3). Moreover, two key studies have indicated that hyperacetylation in histone 3 could increase synaptic plasticity and improve long‐term memory formation. This was proved by the increased LTP and long‐term memory formation after the administration of HDACis.^56^ A prior report is also compatible with our findings showing that amyloid‐beta or ApoE4 increased HDAC translocation to the nucleus, thereby decreasing histone acetylation and BDNF expression.^57^ Contrariwise, ApoE3 promoted histone acetylation and increased BDNF expression. Moreover, with ChIP assay, the synaptic factor is decreased, resulting from the low expression of H3K9Ac and H3K14Ac during prolonged hypoxia. For example, SNAP‐25, which is involved in regulating fusion between the synaptic vesicle and plasma membranes, and its promoter are silenced during hypoxia.^58^, ^59^

Regarding transcription, *BDNF* has 9 exons (I‐IX) in humans and rodents, including eight 5′ non‐coding exons (exons I to VIII) and one 3′ coding exon (Exon IX). Each exon contains its own promoter, resulting in more than 20 transcripts because of alternative splicing and different polyadenylation sites.^60^, ^61^, ^62^, ^63^ How the different *BDNF* mRNA transcripts express temporally and spatially is dependent on its various promoters, which ultimately leads to the regulation of synaptic plasticity and spinal development in dendrites.^64^, ^65^, ^66^, ^67^ A great obstacle to the treatment of degenerative diseases is that the disease has often seriously progressed at the time of discovery, which makes the available treatments less effective. However, therapeutic interventions that act through synaptic repair have a broader window of action time and can therefore be successfully used in the later stages of disease, which offers considerable hope for novel and effective treatment modalities. One study showed that HDAC2 inhibits the *BDNF* exon IV promoter, reduces BDNF expression, and thus terminates the late stage of memory consolidation.^68^ Elsewhere, researchers have found that valproic acid, an HDAC inhibitor, enhances *BDNF* P1 and P4 around histone H4 acetylation and enhances extinction training.^69^ This implies that HDACis could inhibit HDAC2 activity and might enhance *BDNF* promoter expression. We found that compound 13 inhibited HDAC1, 2, 6, and 8 activity and increased lysine residue 14 of histone 3 acetylation around *BDNF* P2 or lysine residue 5 of histone 4 acetylation around *BDNF* P2 and P3 (Figure 4B,G,H). We did not exclude the possibility that other lysine residues of histones could be acetylated or that other HDAC activity could be affected, resulting in other *BDNF* promoter expression. Although *BDNF* P2 and P3 activity was increased, we found that the expression of *BDNF* exons II, III and IX were up‐regulated (Figure 5B,C,E). This may have been caused by exon IX, which is a common coding exon that is conjugated by all other exons. Therefore, our findings suggest an intricate regulatory mechanism for the *BDNF* gene, which provides a new concept for the regulation of BDNF in subcellular locations.

There are some limitations in this study, such as the pharmaceutical therapy of HDACis. This includes questions concerning the capacity of HDACis to pass through the BBB, the effectiveness of HDACis over a given period of time, and the specificity of HDACis. Because there is a BBB, it is worth determining whether these hydroxamic acid‐derived compounds can pass through to the brain. Our data showed that compound 13 increased the acetylation of H3K14 and H4K5 in a CCH brain, implying that this compound can pass through the BBB. Compound 13 should be administrated once every 2 days for a consecutive 30 or 90 days. As it is a complicated procedure, the development of forms of HDACis should be performed with more comprehensive and long‐term studies. Finally, HDACis often inhibit diverse HDAC activity, and they are not specific and may cause side effects. A study to clarify the inhibition of a specific HDAC by HDACis for VaD‐related disease is warranted.

## CONFLICT OF INTEREST

The authors declare no potential conflicts of interest with respect to the research, authorship and/or publication of this article.

## AUTHORS’ CONTRIBUTIONS

Yao‐Ching Fang and Lung Chan designed the experiments and wrote the manuscript. Chin‐I Chen prepared the manuscript figures. Amelia Nur Vidyanti performed the stroke surgeries. Jing‐Ping Liou, Mei‐Jung Lai, Chin‐I Chen, Yong‐Kwang Tu collected and analysed the data. Yao‐Ching Fang, Hsueh‐Yun Lee and Chaur‐Jong Hu contributed in critical revision of the manuscript.

## Data Availability

The data that support the findings of this study are openly available in fig share at https://doi.org/10.6084/m9.figshare.11635317.v1.
